# Isolation of a cytolytic subpopulation of extracellular vesicles derived from NK cells containing NKG7 and cytolytic proteins

**DOI:** 10.3389/fimmu.2022.977353

**Published:** 2022-09-15

**Authors:** Miriam Aarsund, Tuula Anneli Nyman, Maria Ekman Stensland, Yunjie Wu, Marit Inngjerdingen

**Affiliations:** ^1^ Department of Pharmacology, Institute of Clinical Medicine, University of Oslo, Oslo, Norway; ^2^ Department of Immunology, Institute of Clinical Medicine, University of Oslo and Oslo University Hospital, Oslo, Norway

**Keywords:** extracellular vesicles, cancer, granzyme B, NK cells, NKG7

## Abstract

NK cells can broadly target and kill malignant cells *via* release of cytolytic proteins. NK cells also release extracellular vesicles (EVs) that contain cytolytic proteins, previously shown to induce apoptosis of a variety of cancer cells *in vitro* and *in vivo*. The EVs released by NK cells are likely very heterogeneous, as vesicles can be released from the plasma membrane or from different intracellular compartments. In this study, we undertook a fractionation scheme to enrich for cytolytic NK-EVs. NK-EVs were harvested from culture medium from the human NK-92 cell line or primary human NK cells grown in serum-free conditions. By combining ultracentrifugation with downstream density-gradient ultracentrifugation or size-exclusion chromatography, distinct EV populations were identified. Density-gradient ultracentrifugation led to separation of three subpopulations of EVs. The different EV isolates were characterized by label-free quantitative mass spectrometry and western blotting, and we found that one subpopulation was primarily enriched for plasma membrane proteins and tetraspanins CD37, CD82, and CD151, and likely represents microvesicles. The other major subpopulation was enriched in intracellularly derived markers with high expression of the endosomal tetraspanin CD63 and markers for intracellular organelles. The intracellularly derived EVs were highly enriched in cytolytic proteins, and possessed high apoptotic activity against HCT-116 colon cancer spheroids. To further enrich for cytolytic EVs, immunoaffinity pulldowns led to the isolation of a subset of EVs containing the cytolytic granule marker NKG7 and the majority of vesicular granzyme B content. We therefore propose that EVs containing cytolytic proteins may primarily be released *via* cytolytic granules.

## Introduction

NK cells broadly recognize cancer cells based on their recognition of stress-induced ligands and changes in MHC class I expression through a set of activating and inhibitory receptors. Target cells are swiftly killed by cytolytic proteins such as granzymes and perforin released from cytolytic granules within the NK cells, or *via* a slower Fas-FasL dependent pathway (1, 2). Their potent anti-tumor capacities have successfully been exploited in several clinical trials against hematological cancers using either haploidentical or allogeneic NK cells, or CAR-NK cells (3–6). However, NK cell therapies for solid tumors have been more difficult to achieve, partly due to their low ability to infiltrate the tumor (7, 8). NK-cell derived extracellular vesicles (EVs) could circumvent this problem, and previous studies have shown that bulk EVs from NK cells (NK-EVs) contain cytolytic proteins, and can induce apoptosis of diverse tumor cell lines (9–14). The exact mechanism for how tumor cell death is induced by NK-EVs is still unclear, but the EVs appear to express several activating receptors such as DNAM-1 and NKG2D, as well as FasL, that could mediate contact with the tumor cells and induce apoptosis (9, 13, 14).

EVs serve as a biological communication system acting as key mediators of both physiological and pathological processes (15). Several studies have highlighted the potential of EVs in regenerative medicine and in cancer therapy (16, 17). A recent study showed similar efficiency of anti-HER2 CAR-T cells and their derived EVs against HER2+ targets, albeit with a delayed kinetics for the CAR-T EVs (18). Another study showed reduced tumor growth in a mouse model of breast cancer utilizing monocyte-derived EVs loaded with doxorubicin (19). There are still few EV protocols that have entered clinical trials for cancer therapy, but a second generation mature dendritic cell-derived EV product was tested for use in advanced non-small cell lung cancer patients with some effect (20). Together, these studies indicate that EVs have the ability to extravasate and infiltrate solid tumors, and possibly escape the suppressive signals in the tumor microenvironment.

Traditionally, the term EV apply to vesicles with origin from either the plasma membrane (microvesicles or ectosomes), from multivesicular bodies (MVB) within the cell (exosomes), or apoptotic bodies. It has been challenging to precisely discern microvesicles from internally-derived EVs, but proteomics studies have indicated that the tetraspanin CD63 is associated to the internal endosomally-derived EVs, while tetraspanins CD9 and CD81 are more frequently observed on microvesicles (21, 22). With increased knowledge about the heterogeneity of the secreted vesicles, it has become clear that EVs represent a very heterogeneous population. Recent studies suggest that EVs may also originate from compartments other than MVBs, including mitochondria and amphisomes (23, 24). The specific functions of these unique EV subpopulations are yet to be determined. Interestingly, studies indicate that cytolytic granules in NK cells and cytotoxic T cells contain intraluminal vesicles (25, 26). This opens for the possibility that cells in a context-dependent manner secrete subpopulations of organelle-specific EVs, and suggest that the lytic granules may be an alternative compartment for EV release in cytotoxic cells.

In this study, we aimed to isolate a subpopulation of cytolytic EVs from NK cells that may be exploited for treatment of solid tumors as a more potent EV product compared to bulk EVs. To this end, we fractionated NK-EVs from the human NK-92 cell line *via* either density-gradient ultracentrifugation (DG-UC) or size-exclusion chromatography (SEC). Comparative quantitative label-free proteomic analysis revealed unique protein landscapes of specific EV fractions separated by DG-UC. Specifically, we identified an EV subpopulation bearing the cytolytic granule marker natural killer granule protein 7 (NKG7) that was enriched in cytolytic proteins. Our data thus indicate that NK cells release vesicles that derive from cytolytic granules.

## Materials and methods

### Primary cells and cell lines

Buffy coats from healthy human donors were obtained from the blood bank at Oslo University Hospital according to the Declaration of Helsinki. The study was approved by the South-Eastern Norway Regional Ethical Committee (REK2012-1452). Primary NK cells were enriched using RosetteSep NK cell Enrichment Cocktail according to manufacturer’s protocol (Stemcell Technologies), and B cells were depleted by using anti-CD19 Dynabeads (ThermoFisher Scientific). Final NK cell purity was >90% CD56^+^ and CD3^-^. The NK-92 cell line was obtained from ATCC (CRL-2407), and was cultured in complete RPMI medium (RPMI-1640 containing 20% FBS, 1% penicillin/streptomycin, 1% sodium pyruvate, and 50 mM 2-mercaptoethanol) supplemented with 500 IU/ml human recombinant IL-2 (R&D Systems). The HCT-116 colorectal carcinoma cell line was cultured in cPRMI with 10% FBS, and split every 2-3 days.

### EV isolation by SEC and DG-UC

NK-92 cells (1x10^6^/ml) were cultured with 10 ng/ml human recombinant IL-15, while freshly isolated primary NK cells (2x10^6^/ml) were cultured in 10 ng/ml of IL-12, IL-15 and IL-18 (R&D Systems) in serum-free AIM-V medium for 48 hrs. Culture supernatants were sequentially centrifuged at 400 g for 5 min, 2.000 g for 20 min, and finally ultracentrifuged using Sw41Ti or Sw55Ti rotors at 180.000 g for 4 hrs or for 2 hrs 15 min, respectively. EV pellets were resuspended in 1 ml DPBS and further purified through either density-gradient ultracentrifugation (DG-UC) or size-exclusion chromatography (SEC). For DG-UC, the EV-enriched pellet from ultracentrifugation was fractionated by flotation on iodixanol density gradients. A total of 0.5 ml of EVs in PBS was mixed with 1.5 ml 60% iodixanol (Stemcell Technologies) and laid at the bottom of the tube, and 1 ml layers of 40%, 35%, 30%, 25%, 20%, 15%, 10%, 5%, 0% iodixanol were subsequently overlaid forming a discontinuous gradient. The gradient was ultracentrifuged at 120.000 gavg (SW41Ti, k-factor 143.9, Beckman Coulter, w/o brake) for 16 hrs 15 min. Fractions of 200 µl were collected from the top to bottom and distributed into a 96-well plate for absorbance measurement at 340 nm and density was calculated from an iodixanol standard curve. Next, the fractions with densities <1.01 g/ml, 1.01 - 1.03 g/ml, 1.03 - 1.06 g/ml, 1.06 - 1.09 g/ml, 1.09–1.11 g/ml, 1.11–1.14 g/ml, 1.14–1.16 g/ml, 1.16–1.19 g/ml, 1.19–1.21 g/ml and >1.21 g/ml were pooled to form 10 individual fractions, respectively. The 10 fractions were diluted and washed in PBS with ultracentrifugation at 180.000 gavg for 2 hrs 15 min (SW55Ti, k-factor 48, Beckman Coulter). All centrifugation steps were performed at 4°C. For SEC, 10 ml Sepharose 4B (GE Healthcare) columns were applied with 1 ml EVs in PBS, and 10 fractions á 1 ml were collected based on gravity. Samples containing EVs were concentrated using Amicon Ultra 0.5 mL centrifugal filters (Millipore). The fractionated EV pellets were resuspended in PBS and used fresh for downstream experiments. Protein concentration was measured with the Pierce BCA Protein Assay Kit (ThermoFisher Scientific) according to the manufacturer’s protocol.

### Western blotting

EVs were mixed with 2x SDS sample buffer and boiled at 90°C for 5 min. Samples were run on 12% SDS-PAGE Criterion gels (Bio-Rad) under non-reducing conditions. After transfer onto PVDF membranes (ThermoFisher Scientific), membranes were blocked with 5% dry milk and incubated overnight at 4°C with primary antibodies against CD63 (Ts63) and CD81 (M38) from ThermoFisher Scientific, against perforin (#1001103) and granzyme B (#2103A) from R&D Systems, and against NKG7 (TIA-1) from Beckman Coulter, and against FasL (bs-0216r) from Bioss. Blots were probed with goat anti-rabbit IgG-HRP or goat anti-mouse IgG-HRP (Bio-Rad), and developed by Pierce ECL Western blotting Substrate (ThermoFisher Scientific).

### Nanoparticle tracking analysis

Primary NK-EVs and NK-92 EVs diluted in PBS (1:50) were analyzed using an LM10 nanoparticle tracking analyzer with a 532-laser (Malvern Panalytics). Samples were analyzed under constant flow conditions (flow rate = 20) at 25°C, and 10 × 60 sec videos were captured. Data were analyzed using NTA software with a detection threshold of 5 and bin size 2. For NK-92 EVs three biological replicates were measured, whereas only one biological replicate were measured for the primary NK-EVs.

### Transmission electron microscopy

EV isolates (10 µg) were added on top of a formvar carbon coated cobber grid for 1 hr. The excess fluid was removed by blotting with a filter paper. The grids were rinsed by dipping in PBS 3 times and dried by a filter paper. The grid with EVs were further fixed by adding a drop of 2.5% glutaraldehyde before washing the grids 5 times with distilled water, and contrasted by adding a drop 2% uranyl acetate. Finally, the grids were rinsed quickly with ice-cold 1.8% methyl cellulose and 0.4% uranyl acetate (MC/UA). The grids were air-dried for 20 min and examined with a FEI Tecnai™ 120 kV transmission electron microscope G2 Spirit TEM (FEI, The Netherlands) equipped with a Morada digital camera and RADIUS imagining software.

### IncuCyte analysis of spheroid apoptosis

HCT-116 spheroids were generated by seeding 1000 tumor cells into a Nunclon Sphera round-bottom 96-well plate (ThermoFisher Scientific) in 200 µl cRPMI. On day 3, medium was renewed, and 20 µg bulk NK-92 EVs or 20 µg NK-92 EVs separated by either SEC or DG-UC were added together with 5 µM CellEvent Caspase-3/7 Green Detection Reagent (ThermoFisher Scientific). The spheroids were monitored every hour for 48 hrs in an IncuCyte S3 instrument (Sartorius), and analyzed by ImageJ.

### Proteomic analysis

Triplicate samples of NK-92 EV fractions 5-7 (DG-UC) or fractions 3-5 (SEC) in PBS from three separate experiments were subjected to LC-MS/MS analysis, as well as DG-UC fractions 5-7 from IL-12/15/18-stimulated primary human NK cells from three separate donors. Equal amount of protein was used as input. DG-UC NK-92 samples were analyzed using Protein Aggregation Capture on Microparticles method, described previously (27) followed by protein reduction, alkylation, and trypsin digestion. The resulting tryptic peptides were analyzed using EvoSepOne coupled to a quadrupole–Orbitrap mass spectrometer (QExactive HF, ThermoElectron) using a 15 cm C18 column with 30 samples/day methods.

SEC-separated NK-92 EVs or DG-UC-separated EVs from primary NK cells were lysed with 0.1% ProteaseMax Surfactant, after which the proteins were reduced, alkylated and digested into peptides with trypsin. The resulting peptide mixture was purified by STAGE-TIP method using a C18 resin disk (3M Empore) before the samples were analyzed by a nanoLC-MS/MS using nanoElute coupled to timsTOF fleX (Bruker) with 60 min separation gradient and 25 cm Aurora C18 column.

MS raw files were submitted to MaxQuant software version 1.6.17.0 (for NK-92 EVs) or version 2.0.3.0 (for primary NK-EVs) for protein identification and label-free quantification (28). Carbamidomethyl (C) was set as a fixed modification and acetyl (protein N-term), carbamyl (N-term) and oxidation (M) were set as variable modifications. First search peptide tolerance of 20 ppm and main search error 4.5 ppm were used. Trypsin without proline restriction enzyme option was used, with two allowed miscleavages. The minimal unique and razor peptides number was set to one, and the allowed FDR was 0.01 (1%) for peptide and protein identification. Label-free quantitation was employed with default settings. UniProt database with “Human” entries (2020) was used for the database searches. The MaxQuant data was further filtered in Perseus (ver 1.6.15.0). First, known contaminants as provided by MaxQuant and reversed entries were excluded, and intensity data was log10 transformed. Then, a criterion of a protein being detected in at least 2 of 3 biological replicates in at least one of the EV fractions was applied. The mass spectrometry proteomics data have been deposited to the ProteomeXchange Consortium *via* the PRIDE (29) partner repository with the dataset identifiers PXD034603 and PXD035977. Data visualization was done using FunRich version 3.1.4 (30) and GraphPad. Assignment of proteins as organelle markers was performed by matching against the UniProt subcellular localization database.

### Immunoaffinity capture of EV subpopulations

Antibodies for immunoaffinity capture including control IgG1 mAb (113-1, Millipore #MAB1273), CD63 (Ts63, ThermoFisher Scientific), CD81 (M38, ThermoFisher Scientific) and NKG7 (TIA-1, clone 2G9, mAb IgG1, Beckman Coulter) were pre-coupled 20 min at room temperature to Pan Mouse IgG Dynabeads (ThermoFisher Scientific) at 4 µg antibody/100 µl of beads. Beads were washed three times with PBS/0.1% BSA. Fractions 5, 6 and 7 separated by DG-UC were mixed with 20 µl of antibody-coupled beads and incubated on rotor at 4°C overnight. EVs were sequentially captured with control IgG, followed by CD81, CD63, and finally NKG7 antibodies. Beads were washed three times with PBS/0.1% BSA prior to analysis. Each pulldown step was repeated once to ensure depletion prior to the next step. The final flow-through was concentrated by Amicon Ultra 0.5 mL centrifugal filters (Millipore) to a volume of 20 µl. The samples were analyzed by Western blotting.

### Statistical analysis

All data are expressed as mean ± SEM of at least three independent experiments using GraphPad Prism. Statistical analysis were performed using non-parametrical Mann Whitney t-test.

## Results

### Density gradient ultracentrifugation separates an EV fraction enriched in cytolytic proteins

**Figure 1:**
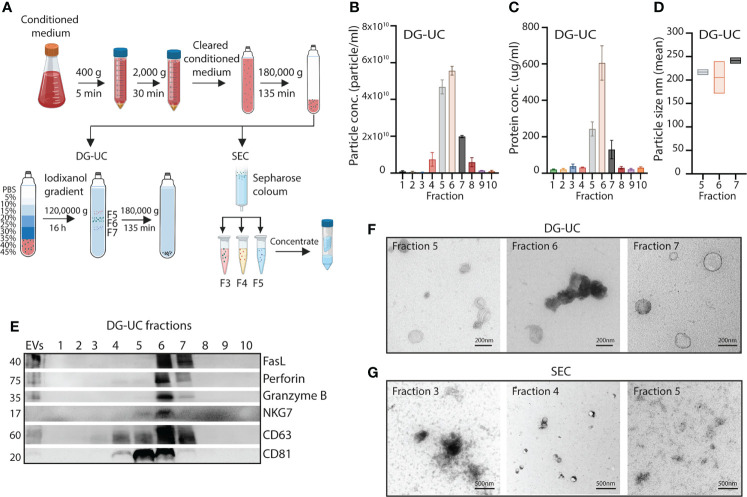
Fractionation of subpopulations of NK-EVs *via* DG-UC or SEC. **(A)** Workflow for isolation of NK-EVs from 48 hrs conditioned medium of IL-15 stimulated NK-92 cells. Cleared conditioned medium was ultracentrifuged, and followed by either DG-UC or SEC as described in Materials and methods. **(B)** Particle concentration of DG-UC fractions 1-10 measured by NTA. Data are presented as mean ±SEM of 3 independent experiments. **(C)** Protein concentration of DG-UC fractions 1-10 measured by micro BCA. Data are presented as mean ±SEM of 3 independent experiments. **(D)** Particle size of DG-UC fractions 5-7 measured by NTA. Data are presented as mean ±SEM of 3 independent experiments. **(E)** Western blot analysis using 20 µg of EV isolates and bulk EVs of NK-92 cells. Representative of three independent experiments. **(F)** TEM of DG-UC subpopulation 5-7 (scale bar 200 nm) and **(G)** TEM of SEC subpopulation 3-5 (scale bar 500 nm).

An overview of the workflow of EV separation is shown in **Figure 1A**. Briefly, NK-92 cells were cultured for 48 hrs in serum-free medium supplemented with IL-15, and conditioned medium was sequentially centrifuged at 400 g and 2,000 g to remove cells and cell debris, followed by bulk EV enrichment *via* ultracentrifugation at 180,000 g for 2 hrs 15 min. Pelleted EVs were further separated into 10 fractions *via* either DG-UC on an iodixanol gradient or *via* SEC as outlined in the methods section.

For EVs separated by DG-UC the highest particle concentrations were observed for fractions 5 and 6, followed by fraction 7 (**Figure 1B**). Protein concentration was highest in fraction 6, followed by fractions 5 and 7 (**Figure 1C**). The size of the particles were mainly found in the range of 100-250 nm, indicating isolation of small EVs (**Figure 1D**). Interestingly, the classical small EV marker CD63 was expressed mainly in fraction 6 of DG-UC EVs, while the EV marker CD81 was strongly detected in both fraction 5 and 6, suggesting existence of distinct vesicles in these two fractions (**Figure 1E**). Less expression of either marker was detected in fraction 7. Testing for perforin, and granzyme B revealed preferential localization within fraction 6, while FasL was found in both fraction 6 and 7. The granule marker NKG7 was mainly localized in fraction 6. The blot was based on EVs isolated from similar volumes of culture supernatant. When blotting with equal protein concentrations in fractions 5-7, we found the same patterns with exception of FasL that appeared enriched in fraction 6 (**Supplementary Figure 1**). We thus conclude that fractions 6 is enriched for EVs containing cytolytic proteins.

TEM analysis indicated morphologically different small EVs within the three fractions (**Figure 1F**). Larger cup-shaped EVs were observed in fraction 5, compared to more electron-dense cup-shaped vesicles in fraction 6. Fraction 7 were enriched for EVs with more translucent EVs, indicating a separate subpopulation of EVs. We previously reported enrichment of CD63, CD81, granzyme B and perforin in fraction 4 of SEC-separated EVs (14). TEM analysis demonstrated similar morphology of vesicles separated by SEC (**Figure 1G**). The divergent profile obtained *via* DG-UC was not captured by SEC, as could be expected as SEC will not separate subpopulations of small EVs to the same extent as DG-UC.

### The cytolytic EV subpopulations efficiently target cancer spheroids

**Figure 2:**
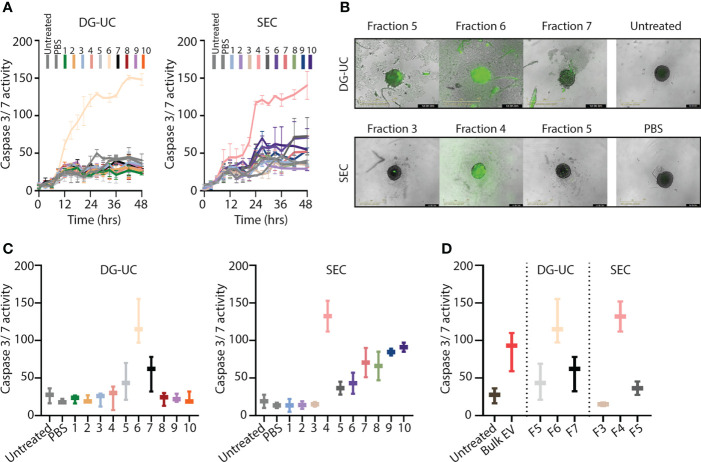
Separation of a subpopulation of NK-EVs that induce apoptosis of HCT-116 tumor spheroids. **(A)** Live monitoring of apoptosis induction *via* IncuCyte by 20 µg NK-EVs from either DG-UC or SEC fractions measured every third hour for 48 hrs. Apoptosis was detected with Caspase 3/7 green detection reagent. Data represents 3 independent experiments ±SEM **(B)** Representative images of HCT-116 tumor spheroids 24 hrs after application of either DG-UC fractions 5-7 or SEC fractions 3-5 imaged by IncuCyte. PBS (in same volume as EVs) or medium alone (untreated) served as negative controls. **(C)** Comparison of apoptosis exerted by 20 µg NK-EVs from DG-UC or SEC fractions after 24 hrs. Data represents 3 independent experiments ±SEM. **(D)** Comparison of apoptosis mediated by 20 µg for either bulk EVs or DG-UC fractions 5-7 or SEC fractions 3-5 after 24 hrs as measured by IncuCyte. Data are presented as mean ±SEM of three separate experiments.

We and others have shown that bulk NK-EVs induce tumor cell apoptosis (9–14). We hypothesized that the fractionation process would enrich for cytolytic EVs, and therefore next compared apoptosis induced by EVs within the different fractions obtained by DG-UC or SEC. Apoptosis of HCT-116 spheroids was tested by live monitoring of Caspase 3/7 activity in spheroids *via* an IncuCyte over 48 hrs using 20 µg EVs as input. Corroborating the Western blotting data of cytotoxic proteins, DG-UC EV fraction 6 and SEC EV fraction 4 uniquely induced apoptosis (**Figure 2A**). For DG-UC fraction 6, HCT-116 apoptosis was evident already at 12 hrs (**Figure 2A**, left panel), while the effect of SEC fraction 4 was delayed. However, at 24 hrs we observed comparable HCT-116 spheroid apoptosis between the two fractions (**Figures 2B, C**). Of note, we observed HCT-116 spheroid apoptosis in SEC fractions 7-10, which could derive from soluble cytolytic proteins eluting in these fractions. Finally, we compared the effect of equal input of bulk EVs with EVs derived from fraction 6 (DG-UC) or fraction 4 (SEC), and found that the fractionated EVs were slightly more effective at inducing spheroid apoptosis compared to bulk EVs (**Figure 2D**). Notably, DG-UC F5 and F7 and SEC F3 and SEC F5 induced negligible apoptosis, underscoring the feasibility of enriching for cytolytic EVs in one single fraction.

### Proteomic profiling of the EV subpopulations reveal unique characteristics

**Figure 3:**
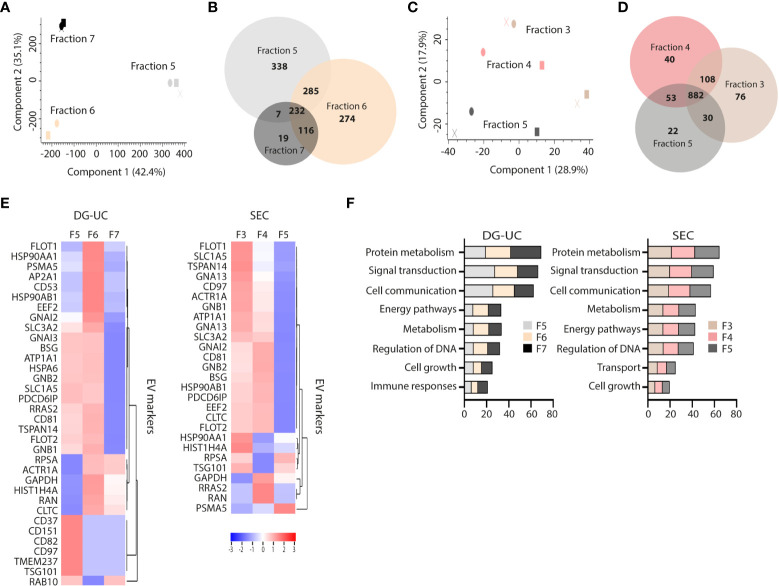
Comparative proteomic profiling of NK-EV subpopulations generated by DG-UC or SEC. **(A)** PCA analysis and **(B)** Venn diagram depicting differences in protein profiles between DG-UC fractions 5-7. **(C)** PCA analysis and **(D)** Venn diagram depicting differences in protein profiles between SEC fractions 3-5. **(E)** Heatmaps of EV markers in DG-UC fractions 5-7 or SEC fractions 3-5 represented by log2 mean intensity values of three biological replicates. **(F)** FunRich biological pathway analysis of proteins identified DG-UC fractions 5-7 or SEC fractions 3-5, showing percentage enrichment of proteins.

The differential profile and function of the EV subpopulations prompted us to a comparative proteomic profiling of the different fractions. Three biological replicates of fractions 5-7 from DG-UC and fractions 3-5 from SEC were subjected to quantitative label-free proteomic analysis. A total of 1271 proteins were reproducibly identified across the DG-UC samples and 1211 proteins across the SEC samples (**Supplementary Tables 1**, **2**, respectively). Principal component analysis of DG-UC EV fractions 5-7 demonstrated a distinctive protein profiles of the three fractions (**Figure 3A**), which was also evident from the Venn diagram comparing the protein identification results from different fractions (**Figure 3B**). Of note, fraction 7 contained fewer identified proteins compared to fractions 5 and 6, and few unique hits. As could be expected, there were considerable more overlap of identified proteins between SEC fractions 3-5 as evidenced by the PCA analysis (**Figure 3C**), where the majority of detected proteins were shared between the three fractions (**Figure 3D**). We matched a recently published EV marker set based on a broad panel of human cell lines (31) against our own dataset, and observed broad distribution of the reported EV markers across the three SEC fractions, while the majority of markers were mainly identified in DG-UC fractions 5 and 6 (**Figure 3E**). Of note, CD81 was identified in DG-UC fractions 5 and 6, confirming the Western blot data (**Figure 1D**). Biological process analysis *via* FunRich revealed that proteins identified within DG-UC fraction 5 and 6 are mainly involved in signal transduction and cell communication, while there was a skewing towards proteins involved in metabolism for DG-UC fraction 7 (**Figure 3F**). No apparent skewing was observed with the SEC fractions (**Figure 3F**). In summary, the DG-UC fractions shows a clear skewing of the proteome indicating presence of EVs with different functions and origins.

### Isolation of an EV subpopulation enriched in NKG7 and cytolytic proteins

**Figure 4:**
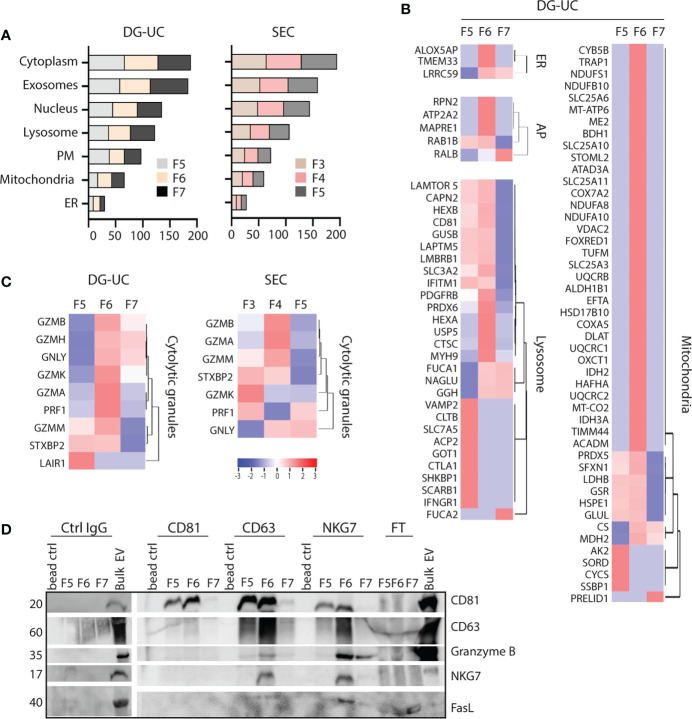
Identification of organelle specific signatures within DG-UC subpopulations. **(A)** FunRich cellular component analysis of proteins identified DG-UC fractions 5-7 or SEC fractions 3-5, showing percentage enrichment of proteins. **(B)** Heatmaps representing 3 three biological replicates of DG-UC fractions 5-7, represented by log2 mean intensity values of mitochondria, endoplasmic reticulum (ER), autophagosome (AP), and lysosome. Markers were identified through matching against the UniProt subcellular localization database. **(C)** Heatmaps representing three biological replicates of DG-UC fractions 5-7 or SEC fractions 3-5, represented by log2 mean intensity values of cytolytic granule markers identified through matching against the UniProt subcellular localization database. **(D)** Representative western blot analysis of sequential immunoprecipitations of DG-UC fractions 5-7 with controls IgG mAb, followed by antibodies against CD81, CD63 and NKG7. EVs from NK-92 cells were used as positive control. FT, flow-through; remaining sample after indicated pulldowns.

Small EVs secreted from cells classically derive from MVBs, but there is evidence in the literature that small EVs also may derive from other intracellular organelles, such as mitochondria (mitovesicles) or cytolytic granules (23, 25). FunRich analysis indicated similar enrichment of proteins belonging to either cytoplasma, exosomes, nucleus, lysosome, plasma membrane, mitochondria or endoplasmatic reticulum (ER) of DG-UC fractions 5-7 or SEC fractions 3-5 (**Figure 4A**). For both fractionation methods, nucleus and lysosomes were the organelles with highest enrichment of proteins in NK-EVs in general. For a more refined analysis, we matched the identified proteins in our dataset against lists of organelle proteins obtained from the UniProt knowledgebase (**Supplementary Table 3**). It was clear from this analysis that mitochondrial, ER and autophagosome proteins predominantly segregated into DG-UC fraction 6 (**Figure 4B**), while lysosomal proteins were identified in a partially non-overlapping manner in DG-UC fraction 5 and 6. The same analysis for SEC-fractions revealed quite heterogeneous identifications across all three fractions (**Supplementary Table 2**). Importantly, cytolytic proteins were highly enriched in DG-UC fraction 6, where the main apoptotic function is observed, but with some overlap with DG-UC fraction 7 (**Figure 4C**). Again, the identified cytolytic granule proteins were more spread across the three SEC-fraction, but notable Granzyme B and A were mainly identified in SEC fraction 4.

Considering the segregation of cytolytic proteins into DG-UC fraction 6, we next tested whether we could enrich for a subpopulation of EVs containing the cytolytic proteins. For this, we made use of NKG7 as a marker for putative cytolytic EVs. NKG7 is specifically expressed in cytolytic granules of NK cells and cytotoxic T cells (32), and shown to play a central role in exocytosis of cytolytic granules (32, 33). We performed sequential immunoprecipitations of EVs in DG-UC fractions 5-7 individually. Assuming that CD81 is contained in both microvesicles and exosomes, we first captured CD81^+^ EVs, followed up by capture of CD63^+^ EVs, and finally NKG7^+^ EVs. The data demonstrate presence of CD81 single positive EVs as well as CD81/CD63 double positive EVs within both fraction 5 and 6. NKG7 was detected both in CD63 and NKG7 pulldowns, indicating the presence of CD63/NKG7 double positive vesicles. Importantly, granzyme B and FasL were mainly localized to NKG7^+^ vesicles pulled down from fraction 6 (**Figure 4D**). This indicates that NK cells release a subpopulation of EVs with cytolytic proteins marked by NKG7.

### Primary NK-EV subsets show similar characteristics as NK-92 derived EVs

**Figure 5:**
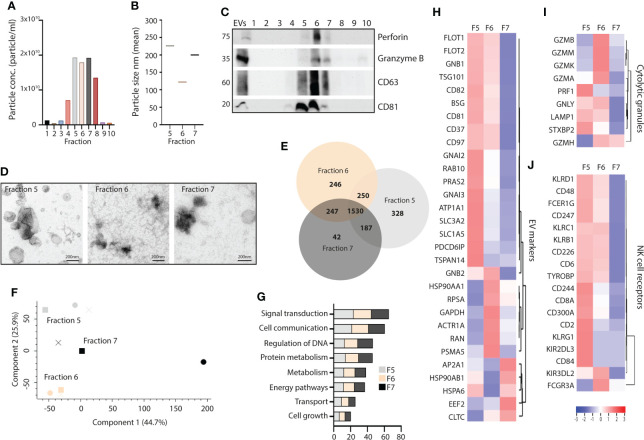
Comparison of fractionated subpopulations of primary NK-cell derived EVs *via* DG-UC. **(A)** Particle concentration of DG-UC fractions 1-10 measured by NTA. Data representing one biological experiment. **(B)** Particle size of DG-UC fractions 5-7 measured by NTA. Data representing one biological experiment. **(C)** Western blot analysis using 20 µg of EV isolates and whole cell lysate (wcl) of primary NK cells. Representative of three independent experiments. **(D)** TEM of DG-UC subpopulation 5-7. Scale bar 200 nm. **(E)** Venn diagram and **(F)** PCA analysis depicting differences in identified protein profiles between DG-UC fractions 5-7 by mass spectrometry. **(G)** FunRich biological pathway analysis of proteins identified DG-UC fractions 5-7 by mass spectrometry. **(H)** Heatmaps representing 3 three biological replicates of DG-UC fractions 5-7, represented by log2 mean intensity values of EV markers, **(I)** cytolytic granules markers and **(J)** NK cell receptors.

Finally, we tested whether we would find the same EV subset profile in primary NK cells, and isolated EVs by DG-UC from primary NK cells cultured in IL-12, IL-15 and IL-18. NTA analysis demonstrated the highest particle counts in fractions 5-7, followed by fraction 8 (**Figure 5A**). The particles sizes was comparable to NK-92 EVs (**Figure 5B**). Importantly, perforin, granzyme B and CD63 were enriched in fraction 6 as observed for NK-92 EVs, while CD81 again was equally distributed between fractions 5 and 6 (**Figure 5C**). The morphology of the primary NK-EV subsets confirmed cup-shaped morphology of EVs in all three fractions (**Figure 5D**). For a deeper phenotyping of the primary NK-EV subsets, we subjected EVs from fraction 5-7 collected from three separate donors to quantitative label-free proteomic analysis. 2295 proteins were detected in fraction 5, 2273 proteins in fraction 6, and 2206 proteins in fraction 7 (**Supplementary Table 4**). There were more overlap observed between the fractions obtained from primary NK cells compared to NK-92 cells (**Figure 5E**), and relatively few unique proteins in fraction 7. This was also illustrated in the PCA analysis, demonstrating that while the different fractions cluster together, they are not well separated (**Figure 5F**). Biological process analysis showed enrichment of proteins involved in signal transduction and cellular communication across the three fractions (**Figure 5G**). As for NK-92 DG-UC EVs, EV markers were preferentially found in fractions 5 and 6, and found at much lower intensities in fraction 7 (**Figure 5H**). Cytotoxic proteins were enriched in fraction 6 with some overlap with fraction 5 (**Figure 5I**), while the majority of NK cell receptors and adaptor proteins were enriched in fraction 5 (**Figure 5J**). Overall, the data thus support that also primary NK cells release a cytolytic EV subpopulation.

## Discussion

EVs possess a major clinical potential in cancer therapy due to their small size, low immunogenicity, relative ease of manipulation of cargo, and stability. In order to fully exploit cytolytic NK-EVs as a therapeutic option, a full understanding of the heterogenic EV secretome from NK cells is needed in order to produce and isolate an optimal product. To this end, we set out to fractionate bulk NK-EVs in order to enrich for cytolytic EVs, and report here the first full characterization of EV subpopulations derived from NK cells by utilizing DG-UC or SEC as an approach to enrich cytolytic EVs, downstream of ultracentrifugation.

In light of recent research, it has become clear that there is a large diversity of EVs released from within the cells. Although the majority of the intracellularly derived EVs (exosomes) are released from MVBs, it is likely that exosomes also may origin from other intracellular compartments. There has been intense efforts to define markers to distinguish microvesicles, or ectosomes, from internally derived EVs, and subpopulations thereof (21, 22). Our dataset largely matches current definition of microvesicles versus exosomes, in that we find CD63 mainly present in putative internally-derived EVs (DG-UC fraction 6), while CD81 is also found in DG-UC fraction 5 that likely represent microvesicles. The latter fraction was enriched in plasma membrane proteins, such as NK cell receptors, as well as proximal signaling molecules, further lending support to DG-UC fraction 5 as mainly microvesicles. Furthermore, the microvesicle/ectosome marker CD99 (22), was exclusively detected in DG-UC fraction 5 with our mass spectrometry analysis, further confirming the enrichment of microvesicles within this fraction. Interestingly, we previously showed that the NK cell line KHYG-1 predominantly released EVs containing CD81 and not CD63, and that these vesicles contained low amounts of cytolytic proteins and lacked in killing activity towards tumor targets (14). These findings support the view that CD81 is present on two distinct vesicle subtypes, where CD81^+^ EVs may derive mainly from the plasma membrane, whereas a distinct population of CD81^+^CD63^+^ vesicles is predominantly endosomally derived.

DG-UC fractions 5 and 6 were both enriched for markers from other cellular compartments, such as mitochondria, nucleus, and endosomally derived proteins. DG-UC fraction 5 therefore also likely contains EVs derived from intracellular sources, although some of the detected markers also may translocate to the plasma membrane. DG-UC fraction 7 contained vesicular structure as observed by electron microscopy, but we found little expression of EV markers or tetraspanins in this fraction by either western blotting or proteomics. Particle concentration was quite low, so it remains to be determined what these vesicles represent. However, we did detect an enrichment of proteins involved in metabolism in this fraction.

Although we detected strong signals for CD63 by Western blotting, we found little or no CD63 by mass spectrometry analysis in EVs from either NK-92 or primary NK cells. This matches immunogold labeling of primary NK cells by electron microscopy, where CD63 is surprisingly scantly detected, and mainly in MVBs (data not shown). Of note, we found that the EVs in fraction 5 and 6 from both NK-92 and primary NK cells contain a number of other tetraspanins, in particular fraction 5 was enriched for CD37, CD82 and CD151, while NK-92 EV fraction 6 contained CD53. The specific functional role of these tetraspanins for NK-EVs is not known, but both CD53 and CD151 are reportedly involved in controlling adhesion and migration (34, 35), and CD151 may sort integrins into EVs (36).

The potential for using NK-EVs in cancer therapy is obvious, and several studies have previously demonstrated that NK-EVs exert cancer-specific cytotoxicity without affecting primary human cells (9, 11, 37). Recently, we showed that NK-EVs exert a cytotoxic activity against a range of solid tumor cell lines, including WM-9, HCT-116 and T-4D7 (14). However, studies demonstrating anti-tumor activity of NK-EVs have focused on bulk EVs. Isolating a more pure population of cytolytic EVs that exclusively contains cytotoxic proteins, could be more potent as a future therapeutic EV-therapy compared to bulk EVs. Importantly, when we compared bulk EV to the cytolytic fraction 6 (DG-UC) and fraction 4 (SEC), both induced enhanced tumor cell apoptosis compared to bulk EVs.

Ultracentrifugation currently remains the international standard for EV isolation, despite drawbacks such as insufficient separation of contaminating proteins and being time-consuming (38), but combined with DG-UC or SEC a purer EV isolate is achieved. Although ultracentrifugation followed by DG-UC yields a relatively pure EV isolate, it is a time consuming protocol with relative low yields. While SEC is a less labor-intensive protocol than DG-UC, fractionation *via* SEC is too crude to allow fine separation of EV subpopulations. Regardless, we show an enrichment of cytolytic proteins within one SEC-fraction, demonstrating that SEC may be useful for separating functional, cytolytic EVs from soluble proteins. For clinical applications, it may therefore be more feasible to apply SEC as separation protocol, perhaps linked to downstream immunoaffinity isolation of cytolytic EVs.

We show here that cytotoxic proteins stored in NK cell cytotoxic granules including different types of granzymes, granulysin, and perforin were almost exclusively found within fraction 6 (DG-UC) and fraction 4 (SEC) in NK-92 EVs. This was also recapitulated using primary human NK-cell derived EVs. We further showed that we from DG-UC fraction 6 could pull out NKG7^+^ vesicles that were enriched in granzyme B and FasL. The tetraspanin NKG7 is expressed by NK cells and cytotoxic CD8^+^ T cells, and is an essential mediator for granule-mediated target cell death (33). Our data thus suggest that there exist intraluminal vesicles that are released from cytolytic granules. In support of this notion, recent studies have indicated that the E3 ubiquitin ligase NKLAM (Natural Killer lytic-associated molecule), which is expressed in the membrane of cytolytic granules of NK cells, can be detected also in the membranes of bulk EVs (39). Also, a recent study identified T-cell derived vesicles that were released in context of an immunological synapse between the T cells and antigen presenting cells, and that were enriched in effector proteins (40). We did not detect NKG7 by mass spectrometry, and speculate that it may be expressed at low levels in NK cells, or difficult to detect akin to CD63. Cytokine stimulation greatly enhance the cytotoxic capacity of NK cells and expression of cytolytic proteins, and it will be important in the future to fully characterize how different activation protocols, such as triggering though activating receptors, may impact the release of subsets of cytolytic EVs. Moreover, cytokine cultures alone will not trigger a major release of cytolytic granule content, therefore the release mechanism of cytolytic EVs in response to cytokines needs further investigation.

In conclusion, we have performed a comparative profiling of EV subpopulations isolated *via* either DG-UC (NK-92 or primary NK cells) or SEC (NK-92 cells), and show that we can separate the bulk of plasma membrane derived EVs from EVs derived from intracellular sources. Induction of tumor cell apoptosis was only observed with the EVs of intracellular origin, and not with EVs derived from the plasma membrane. Protocols to further increase the yield of the cytolytic EVs could be instrumental for their exploitation in cancer therapy.

## Data availability statement

The data presented in the study are deposited in the PRIDE repository, accession numbers PXD034603 and PXD035977.

## Author contributions

MA conducted experiments, analysed data, wrote the manuscript, MS conducted experiments and analysed data, TN analysed data and contributed to the manuscript, YW conceived the study, conducted experiments, analysed data and edited the manuscript, MI conceived the study, analysed data and edited the manuscript. All authors contributed to the article and approved the submitted version.

## Funding

Our study was supported by the Research Council of Norway NANO2021-program (grant number: 303256). Mass spectrometry-based proteomic analyses were performed by the Proteomics Core Facility, Department of Immunology, University of Oslo/Oslo University Hospital, which is supported by the Core Facilities program of the South-Eastern Norway Regional Health Authority and a member of the National Network of Advanced Proteomics Infrastructure (NAPI), funded by the Research Council of Norway INFRASTRUKTUR-program (project number: 295910).

## Acknowledgments

The authors would like to thank the Electron Microscopy Core Facility at Oso University Hospital.

## Conflict of interest

The authors declare that the research was conducted in the absence of any commercial or financial relationships that could be construed as a potential conflict of interest.

## Publisher’s note

All claims expressed in this article are solely those of the authors and do not necessarily represent those of their affiliated organizations, or those of the publisher, the editors and the reviewers. Any product that may be evaluated in this article, or claim that may be made by its manufacturer, is not guaranteed or endorsed by the publisher.
