# Supplementary figures and images for: Isolation of a cytolytic subpopulation of extracellular vesicles derived from NK cells containing NKG7 and cytolytic proteins
Source: Front Immunol. 2022 Sep 15;13:977353. doi: 10.3389/fimmu.2022.977353 (PMC9520454; doi:10.3389/fimmu.2022.977353)

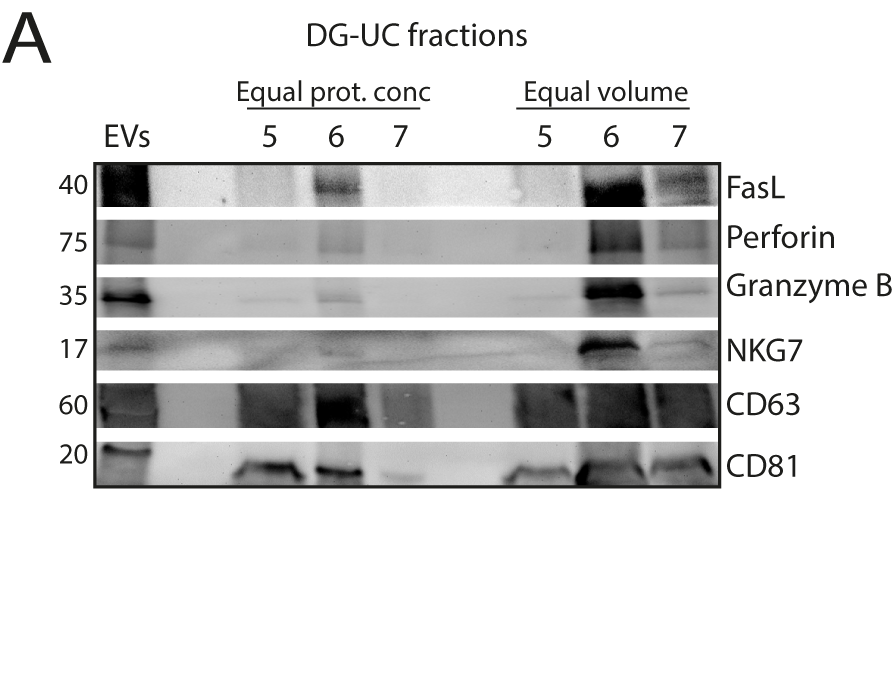

Supplement: Supplementary file 1 [file Image_1.tif]
